# Diet in Lithemia

**Published:** 1900-01

**Authors:** A. B. Conklin


### DIET IN LITHEMIA.

BY A. B. CONKLIN, M.D.

With those who, like the philosopher of old, "ate to live instead of living to eat," the taking of food is for the purpose of maintaining the nutrition of the body tissues and securing the functional activity of every organ and part.

While this can be done more or less perfectly with a great variety of foods, yet observation and experiment have demonstrated that any article of food, or combination of foods, to accomplish this end, must contain both albuminous and carbonaceous constituents in something like definite proportions.

Though these proportions may be varied somewhat in different latitudes, and in different individuals under different conditions of environment, yet it is accepted as a general rule that an ideal diet should contain about seven parts of carbonaceous constituents to one of albuminous, the former to supply heat to the body, the latter to furnish force, energy, strength, and endurance.

Again, since the preservation of life, and the maintenance of physiological integrity come only from the ingestion of food, selected with the proper regard for its composition, it follows that any material deviation from this standard of requirement must result in those functional perversions and tissue changes we call disease.

Inversely it follows that while in health food serves the one purpose of maintaining life, we find in many pathological conditions, and especially such as have arisen from an improper diet, that much may be accomplished in restoring the normal conditions by a proper selection of food.

In the management of lithemic cases, which are due to a greater extent to improper food than any other cause, a proper diet is of paramount importance.

The diet of the lithemic resolves itself into (1), the taking of the necessary amount of albuminous and carbonaceous foods to properly nourish the body, and (2), the selection of such albuminous foods as are most easily digested, most perfectly assimilated and converted into urea and leave behind the least amount of uric acid.

Because an albuminous food readily undergoes gastric digestion, it does not necessarily follow that assimilation will be perfect, or that its nitrogenous elements will undergo complete metabolism into urea; hence the problems of selecting the most eligible albuminous food is not solved by the measure of digestibility.

Rare roast beef for instance, which is digested in three hours, is not as readily assimilated and as perfectly metabolized into urea as the albuminous constituents of wheaten bread that requires one half-hour longer to digest.

Furthermore, if we accept the late teachings of Haig on diet, the amount of uric acid resulting from the ingestion of a given amount of albuminous food from animal origin is greater than that resulting from a like amount of albumens from a vegetable source.

Since the deductions he has drawn from his studies in physiological chemistry are in keeping with the results of my own observation, I shall in my endeavor to map out a diet for lithemic patients merely reiterate what I have said many times during the past ten years, viz.: that meat is not a suitable food for the lithemic.

While lard and the other animal fats are products of animal tissue, it is not necessary to consider them in connection with a lithemic diet, for the reason that they are carbonaceous instead of albuminous, and probably have no direct influence upon the elaboration of uric acid.

Another animal food, however, which must not be overlooked, is milk and milk products. A consideration of butter and cream may be omitted as we have done with lard, and for the same reason. Not so with cheese. It is about one-third albuminous matter, and if used at all, should be used very guardedly. It should be taken only in small amounts at any one time, thoroughly masticated and distributed throughout an entire meal of less concentrated foods, like vegetables, cereals and fruits. In extreme cases it may be positively contraindicated.

Another form of animal food to be considered is eggs, with a percentage of albumen almost equal that of lean beef, and showing a digestibility of one and a half hours raw, whipped, and three and a half hours fried or hard boiled.

Except in cases where the uric acid accumulation is already great, raw eggs whipped in milk may be permitted, but fried or hard boiled never.

Coming now to purely vegetable foods, it may be said that almost without exception they are suited to the lithemic, and may be selected with much variety, ever keeping in mind their percentage of albuminous constituents and limiting the use of such as are most highly nitrogenous.

Peas, beans and lentils, because they contain the largest percentage of albumens of all the vegetable foods, should be taken, if at all, but sparingly, and in the same manner and with the same circumspection with which cheese may be taken. All other vegetable foods, including fruits, may be included in the dietary of the lithemic as a rule.

The vegetable foods, which in some cases may have an unpleasant effect, are the pulses and cereals, which tend, like meats, by their acids and acid salts, to keep up the acidity of the urine.

In most cases milk, with whole wheat bread thoroughly baked, rice, oatmeal, barley and rye meal and fruits, will constitute an ideal diet.

The vegetables and fruits may be selected according to the tastes and digestive powers of the patient, and the percentage of albuminous and carbonaceous constituents as shown by the following table:

FOOD TABLE.

NUTRITIVE VALUE OF FOODS IN 100 PARTS.

| | Albuminous. | Carbonaceous. | | Albuminous. | Carbonaceous. |
|---|---|---|---|---|---|
| MEATS.— | | | MEATS.— | | |
| Cheese | 33 | 37 | Veal | 16.5 | 15.8 |
| Poultry | 21 | 3.6 | Salmon | 16.1 | 5.5 |
| Lean beef | 19.3 | 3.6 | Pork | 9.8 | 48.9 |
| Lean muton | 18.3 | 4.9 | New milk | 4.1 | 9.1 |
| White fish | 18.1 | 2.9 | Cream | 2.7 | 29.5 |
| Entire egg | 18 | 10.5 | | | |
| PULSES.— | | | PULSES.— | | |
| Beans | 30.8 | 50.2 | Peas | 23.8 | 60.8 |
| Lentils | 25.2 | 58.6 | | | |
| CEREALS.— | | | CEREALS.— | | |
| Oatmeal | 12.6 | 69.4 | Rye meal | 8 | 75.2 |
| Indian meal | 11.1 | 73.2 | Rice | 6.3 | 80.2 |
| Whole wheat flour | 10.8 | 72.5 | Barley meal | 6.3 | 76.7 |
| Bread | 8.1 | 52.6 | | | |
| VEGETABLES.— | | | VEGETABLES.— | | |
| Potato | 2.1 | 22.2 | Turnip | 1.2 | 7.2 |
| Sweet potato | 1.5 | 27.5 | Parsnip | 1.1 | 15.9 |
| Beet | 1.5 | 11.3 | Cabbage | .9 | 4.1 |
| Carrot | 1.3 | 14.7 | | | |

| FRUITS.— | Albuminous. | Carbonaceous. | FRUITS.— | Albuminous. | Carbonaceous. |
|---|---|---|---|---|---|
| Date | 9 | 58 | Gooseberry | .4 | 8.9 |
| Banana | 4.8 | 20.2 | Peach | .4 | 7.8 |
| Cherry | .9 | 15.3 | Strawberry | .3 | 7.1 |
| Grape | .8 | 14.3 | Apple | .2 | 10.3 |
| Raspberry | .5 | 6.4 | Pear | .2 | 10.2 |
| Blackberry | .5 | 5.8 | Plum | .2 | 9.3 |
| Currant | .4 | 5 | Sugar | | 95 |

No diet, however carefully selected, would fully meet the requirements of the lithemic which did not contain as carefully a selected list of drinks as of foods.

Alcohol meets no need of the lithemic. All spirituous liquors, beer, ale, and sour wines should be strictly denied him.

Tea, coffee, cocoa and other similar alkaloid-bearing substances are not suitable for a lithemic, and should not be permitted.

Water, milk, and the various drinks made from it, fruit juices, clear or with water, Caramel Cereal, and other brands of roasted cereals will furnish an ample variety of wholesome and harmless drinks.

A bright, sparkling cider, not too old, and made from sound fruit, is often an advantageous drink for the lithemic. The proverbial after-dinner cigar should go the way of the alcoholics.

